# Accentuating CircRNA-miRNA-Transcription Factors Axis: A Conundrum in Cancer Research

**DOI:** 10.3389/fphar.2021.784801

**Published:** 2022-01-11

**Authors:** Deepti Singh, Prashant Kesharwani, Nabil A. Alhakamy, Hifzur R. Siddique

**Affiliations:** ^1^ Molecular Cancer Genetics and Translational Research Lab, Section of Genetics, Department of Zoology, Aligarh Muslim University, Aligarh, India; ^2^ Department of Pharmaceutics, School of Pharmaceutical Education and Research, Jamia Hamdard, New Delhi, India; ^3^ Department of Pharmaceutics, Faculty of Pharmacy, King Abdulaziz University, Jeddah, Saudi Arabia

**Keywords:** circrnas, cancer, miRNA sponge, transcription factors, targeted therapy

## Abstract

Circular RNAs (circRNAs) are the newly uncovered class of non-coding RNAs being cognized as profound regulators of gene expression in developmental and disease biology. These are the covalently closed RNAs synthesized when the pre-mRNA transcripts undergo a back-splicing event. In recent years, circRNAs are gaining special attention in the scientific world and are no longer considered as “splicing noise” but rather structurally stable molecules having multiple biological functions including acting as miRNA sponges, protein decoys/scaffolds, and regulators of transcription and translation. Further, emerging evidence suggests that circRNAs are also differentially expressed in multiple cancers where they play oncogenic roles. In addition, circRNAs in association with miRNAs change the expression patterns of multiple transcription factors (TFs), which play important roles in cancer. Thus, the circRNA-miRNA-TFs axis is implicated in the progression or suppression of various cancer types and plays a role in cell proliferation, invasion, and metastasis. In this review article, we provide an outline of the biogenesis, localization, and functions of circRNAs specifically in cancer. Also, we highlight the regulatory function of the circRNA-miRNA-TFs axis in the progression or suppression of cancer and the targeting of this axis as a potential therapeutic approach for cancer management. We anticipate that our review will contribute to expanding the knowledge of the research community about this recent and rapidly growing field of circRNAs for further thorough investigation which will surely help in the management of deadly disease cancer.

**GRAPHICAL ABSTRACT:**
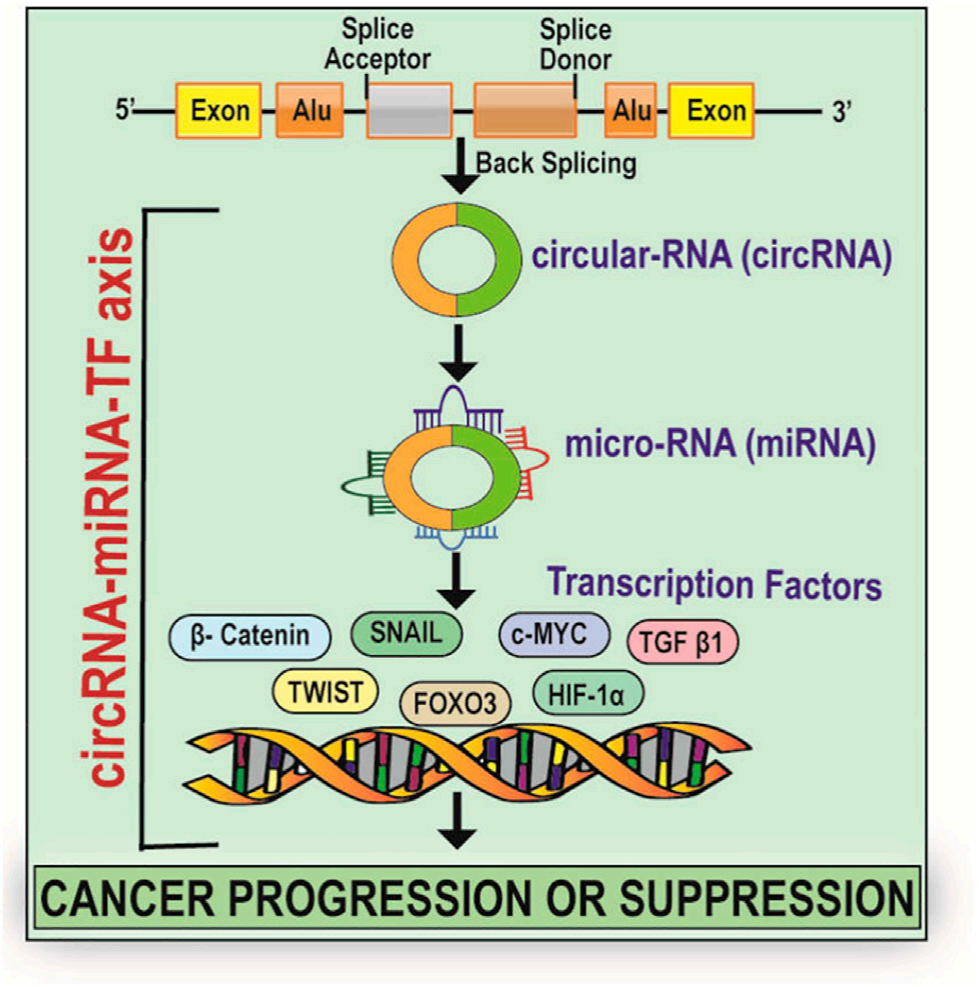


## Introduction

Cancer involves a broad range of dysregulations in the genome, transcriptome, and proteome. Most of the transcriptome studies related to cancer are concentrated mainly on the changes or dysregulations in the protein-coding genes, focusing less on non-coding RNAs (ncRNAs). These ncRNAs were earlier deemed as “junk of transcription”. But recent studies have reported the emerging contribution of non-coding RNAs in various aspects of carcinogenesis (Singh et al., 2020). According to the length of the transcript, ncRNAs are grouped into small ncRNAs (<200) and long ncRNAs (>200) (Uchida and Adams, 2019). Among the ncRNAs, circular RNAs (circRNA) are a group of endogenous ncRNAs, first discovered in 1976 in the genome of viruses and later found in the cytoplasm of eukaryotic cell lines in 1979 (Sanger et al., 1976; Hsu and Coca-Prados, 1979; Kolakofsky, 1976). Since then, circRNAs remained unexplored due to limitations in technology. However, recent technological advancements and the emergence of techniques such as next-generation sequencing and bioinformatics have led to the identification of the abundance and diversity of circRNAs and their different expression patterns in various physiological conditions is becoming a focus of research. CircRNAs are a group belonging to the large class of ncRNAs produced by a non-canonical splicing event called back-splicing (Chen, 2016). The circRNAs are generated in the nucleus but are exported to and localized predominantly in the cytoplasm (Salzman et al., 2012; Jeck et al., 2013). These are formed by the ligation of the 5′ end of the upstream exon and 3′end of the downstream exon or the by joining of ends of individual exons. This covalently closed circular structure of circRNAs makes them stable against RNase digestion as compared to the linear RNAs (Enuka et al., 2016). CircRNAs function at various levels of gene regulation. For instance, at transcriptional, splicing, and translational level *via* acting as miRNA sponge; protein decoys; and by encoding several functional peptides. Recent reports suggest that circRNAs are dysregulated in various diseases including neurological, cardiovascular, immunological diseases, and cancer (Piwecka et al., 2017; Aufiero et al., 2019; Kristensen et al., 2019; Vo et al., 2019; Wang et al., 2019).

Interestingly, the accumulation of circRNAs in non-proliferative cells (nervous tissue) and downregulation of circRNAs in proliferative cells have also been reported. During proliferation, circular and linear RNAs are evenly distributed in the daughter cells. The steady-state levels of linear transcripts are regulated mainly due to active transcription and degradation, also resulting in a new expression of circRNAs and constant ratios of circular to linear RNAs (Bachmayr-Heyda et al., 2015). However, the downregulation of circRNAs in proliferating cells is mainly attributed to the dilution of circRNA concentration upon cell division thus reducing the tumor-suppressive potential of circRNAs (Bachmayr-Heyda et al., 2015; Gruner et al., 2016; Vo et al., 2019). In contrast, in non-proliferating cells, linear transcripts are at a steady rate of transcription and degradation while the circRNAs remain stable and accumulate. This negative correlation observed between the abundance of circRNA and proliferation could be used in positioning some of the circRNAs as proliferation markers (Dahl et al., 2021). Further, the upregulated circRNAs could be used to distinguish specific cancer types and as potential indicators of genomic amplification.

CircRNAs are also dysregulated in several human malignancies and represent distinct expression patterns in various cancer types. They are found to be globally reduced in cancerous tissues as compared to the normal tissues possibly due to the following reasons: the compromised back-splicing machinery in the cancerous tissues; passive thinning of the circRNAs due to cell proliferation and the increased degradation by miRNAs which are deregulated in the cancerous tissues (Bachmayr-Heyda et al., 2015). CircRNAs have also been reported to play tumor-suppressive or oncogenic roles leading to either the suppression or progression of cancer thereby serving as potential therapeutic targets for cancer diagnosis and treatment (Han et al., 2017; Hu et al., 2018; Chen et al., 2019). For instance, circMTO1 is reported to be downregulated in hepatocellular carcinoma (HCC) and cervical cancer (Han et al., 2017; Chen et al., 2019) whereas circPVT1 is found to be upregulated in leukemia (Hu et al., 2018). Further, some of the circRNAs also show inconsistent expression patterns in cancer types. For instance, the circHIPK3 which is transcribed from Exon2 of the HIPK3 gene is upregulated in HCC (Chen et al., 2018a) but it is downregulated in ovarian cancer (Teng et al., 2019). Recent evidence suggests that circRNAs play a significant part in transcriptional control by acting as miRNA sponges and regulating the target genes of miRNAs. miRNAs negatively regulate the expression of the target gene by targeting the 3′UTR of its target mRNA thereby reducing its stability and inhibiting its translation. Thus, several circRNAs have been recently discovered as oncogenic drivers or suppressors of tumorigenesis *via* the circRNA-miRNA-TF axis. In this review, we will highlight the representative studies depicting the regulatory role of the circRNA-miRNA-TF axis in the initiation or suppression of cancer with the aim of suggesting potential implications for both clinical and therapeutic applications.

## Biogenesis and Functions of circRNAs

**FIGURE 1:**
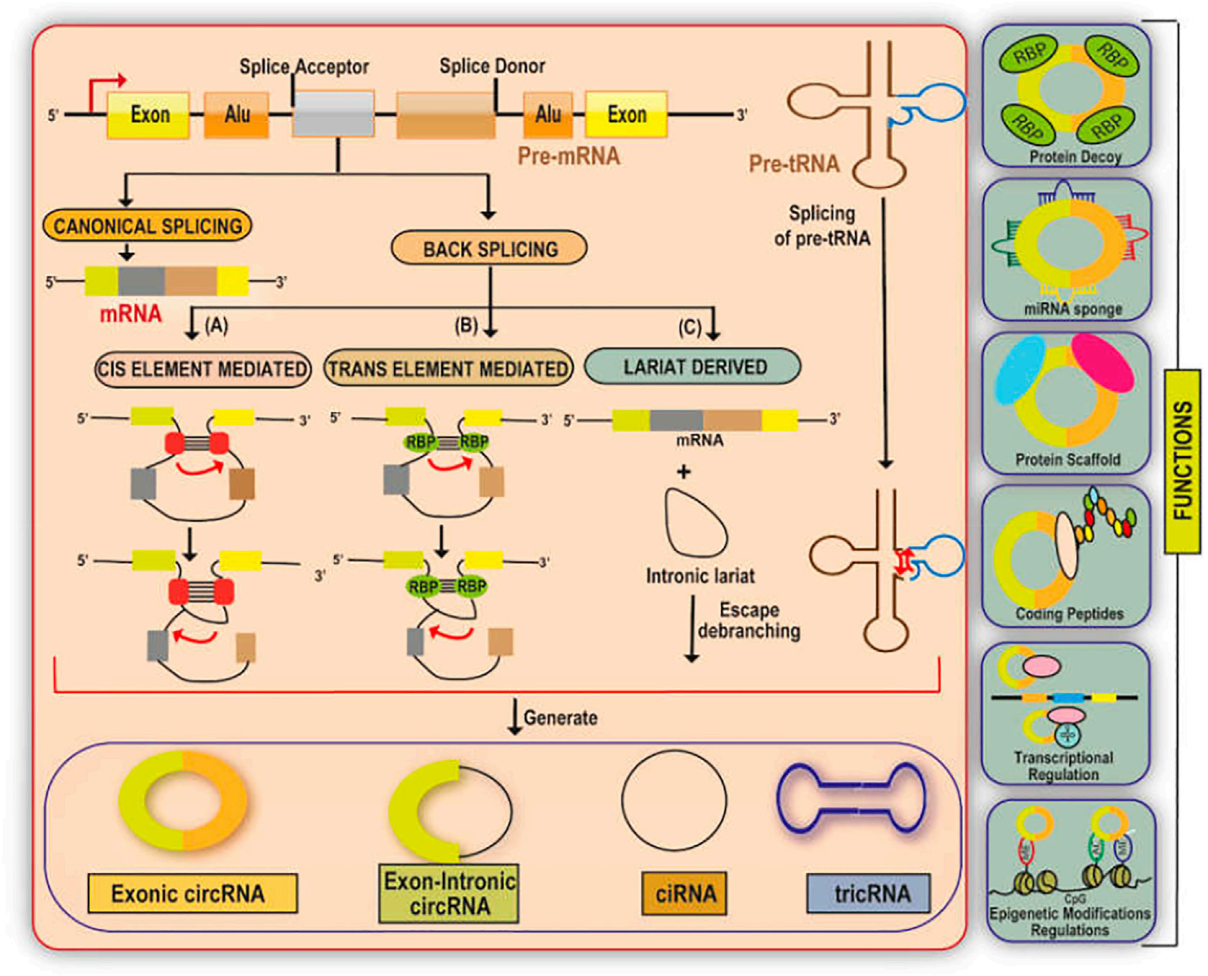
A diagrammatic illustration of the biogenesis and functions of circular RNA. circRNA are synthesized from either pre-mRNA or pre-tRNA. The loop structure in the cis/trans element derived biogenesis of circRNA is mediated by base pairing between the complementary sequences which flank the RBPs or the circularised exons. Further, EcRNA, or EIciRNAs are produced by either removing or retaining the intron sequences in the loop structure respectively. circRNA are also produced *via* lariat derived circularisation with an exon-skipping event. The generation of ciRNA depends on the 7-nt GU sequence and 11-nt C rich sequences present near the 5′ splice site and the branch point site respectively which form a lariat intron. The synthesis of tric RNA involves the presence of an intron-containing pre-tRNA which is cleaved at the BHB motif into an exon half and an intron part. The resulting exons halves are combined to form a mature tRNA and the intron termini form the tricRNA. CircRNAs function mainly as regulatory ncRNAs either by directly regulating the transcription of the gene, by serving as miRNA sponges, protein decoys/dynamic scaffolds, or in the translation of regulatory peptides and regulation of epigenetic modification.

CircRNAs are generated primarily by a non-canonical splicing event called back-splicing, where a downstream splice-donor site is linked covalently to an upstream splice-acceptor site. (Figure 1). According to the source of origin, circRNAs can be classified into exon derived circRNAs (EcRNAs), exon-intron derived circRNAs (EIciRNAs), and intron derived circRNAs (Salzman et al., 2013; Zhang et al., 2013; Li et al., 2015a; Lu et al., 2015). The intron-derived circRNAs are further divided into circular intronic RNAs derived from pre-mRNA (ciRNAs) and tRNA intronic circular RNAs (tricRNAs) (Zhang et al., 2013; Lu et al., 2015; Schmidt et al., 2019). The biogenesis of circRNAs involves spliceosomal machinery which ligates the 5′splice site of an upstream exon to the 3′ splice site of another downstream exon thereby forming a closed structure (Ashwal-Fluss et al., 2014; Starke et al., 2015; Kristensen et al., 2019). This biogenesis is mediated by cis-acting and trans-acting elements. Cis-acting elements function as complementary sequences in the introns which flank the back spliced exons. These cis-acting elements undergo base pairing resulting in the formation of hairpin structure and put forward the 5′splice site present downstream and 3′ splice site present upstream close enough to enable the easy circularisation of circRNAs (Zhang et al., 2014; Chen, 2016; Wu et al., 2019). The trans-acting factors involved in the biogenesis of circRNAs include RNA binding proteins (RBPs) such as Muscleblind (MBL) and Quaking which recognize and bind to particular regions/motifs within the flanking introns and form a bridge connecting the two splice sites *via* protein-protein interaction or self-dimerization thereby strengthening the back splicing of introns (Ashwal-Fluss et al., 2014; Conn et al., 2015).

CircRNAs predominantly function as regulatory ncRNAs either at the transcriptional or translational level (Figure 1). CircRNAs associate directly with the RNA Polymerase II (RNA Pol -II) machinery to stimulate the parent gene transcription (Zhang et al., 2013). For instance, RNA-RNA interactions between ElciRNA and U1 small nuclear ribonucleoproteins (U1 snRNP) result in forming a complex which then interacts with RNA Pol-II thereby enhancing the host gene transcription (Li et al., 2015a). CircRNAs also function as miRNA sponges and prevent the miRNA-regulated suppression of the target gene. For instance, circRNA ciRS-7 binds and sponges miR-7 and AGO protein complex to elevate the expression levels of miR-7 targets (Hansen et al., 2013). Further, a single circRNA may have binding sites for multiple miRNAs such as in the case of circSLC8A1 which can sponge both miR-133 and miR-130b/494 (Lim et al., 2019; Lu et al., 2019a). CircRNAs also function as protein decoys in sequestering the proteins. For example, circPABPN1 acts as a competitor of PABPN1 mRNA to bind with HuR thereby suppressing the PABPN1 translation. HuR is an RNA-binding protein having multiple mRNA targets such as TP53, MYC, and HIF-1α (Abdelmohsen et al., 2017; Singh et al., 2021a). Li et al. (2019) reported that circRNAs harbor binding sites for multiple proteins thereby functioning as dynamic protein scaffolds bringing about protein-protein interactions for assembling large RNA-protein complexes. For instance, circACC1 cohere to the β and γ subunits of AMPK resulting in the formation of a ternary complex, thereby stabilizing and promoting the AMPK enzymatic activity (Li et al., 2019).

Although, circRNAs lack a 5′ m^7^G Cap and 3′ poly (A) tail which are essential for linear transcripts to recruit the initiation factors of translation, circRNAs could undergo cap-independent translation mediated in an m^6^A modification dependent manner or an internal ribosome entry site (IRES) element dependent manner (Legnini et al., 2017; Yang et al., 2017a). Only a few endogenous circRNAs such as circ-ZNF609, circMbl, circ-FBXW7, circPINTexon2, and circ-SHPRH have been reported to function as templates for translation (Legnini et al., 2017; Pamudurti et al., 2017; Yang et al., 2017b; Yang et al., 2018a; Zhang et al., 2018a; Zhang et al., 2018b). In IRES element-dependent manner, IRES recruit the 40S subunit of the ribosome to RNAs in a 5′Cap independent manner and enable the translation of many endogenous circRNAs. m^6^A modification also helps in the translation of circRNAs. For instance, YTHDF3 (a reader protein for m^6^A) binds to the m^6^A containing circRNAs and further interact with the eIF4G2 and eIF3A translation initiation factors to initiate protein synthesis (Yang et al., 2017b). Since, m^6^A modifications are present in several circRNAs, whether or not, all of these m^6^A modified circRNAs having ORFs will be translated in an m^6^A-dependant manner remains difficult to find and studies are underway to explore this issue. Further, most of the circRNA-derived peptides are truncated forms of canonical proteins and lack essential functional domains, thereby acting as the dominant-negative variants of proteins or as modulators of alternative protein complexes (Legnini et al., 2017). Also, circRNAs are expressed at low levels than the corresponding mRNAs making it difficult to determine whether a circRNA is translated or not. The presence of ORF, functional IRES-like element, and m^6^A methylation can be used to assess a translated circRNA using luciferase reporters (Legnini et al., 2017; Pamudurti et al., 2017; Yang et al., 2017a; Yang et al., 2018b; Zhang et al., 2018a; Zhang et al., 2018b). Further, polysome profiling and ribosome footprinting can be used to explore whether a circRNA is translated or not (Zhang et al., 2018c).

## Aberrantly Expressed CircRNAs in the Progression of Various Cancer Types

**FIGURE 2:**
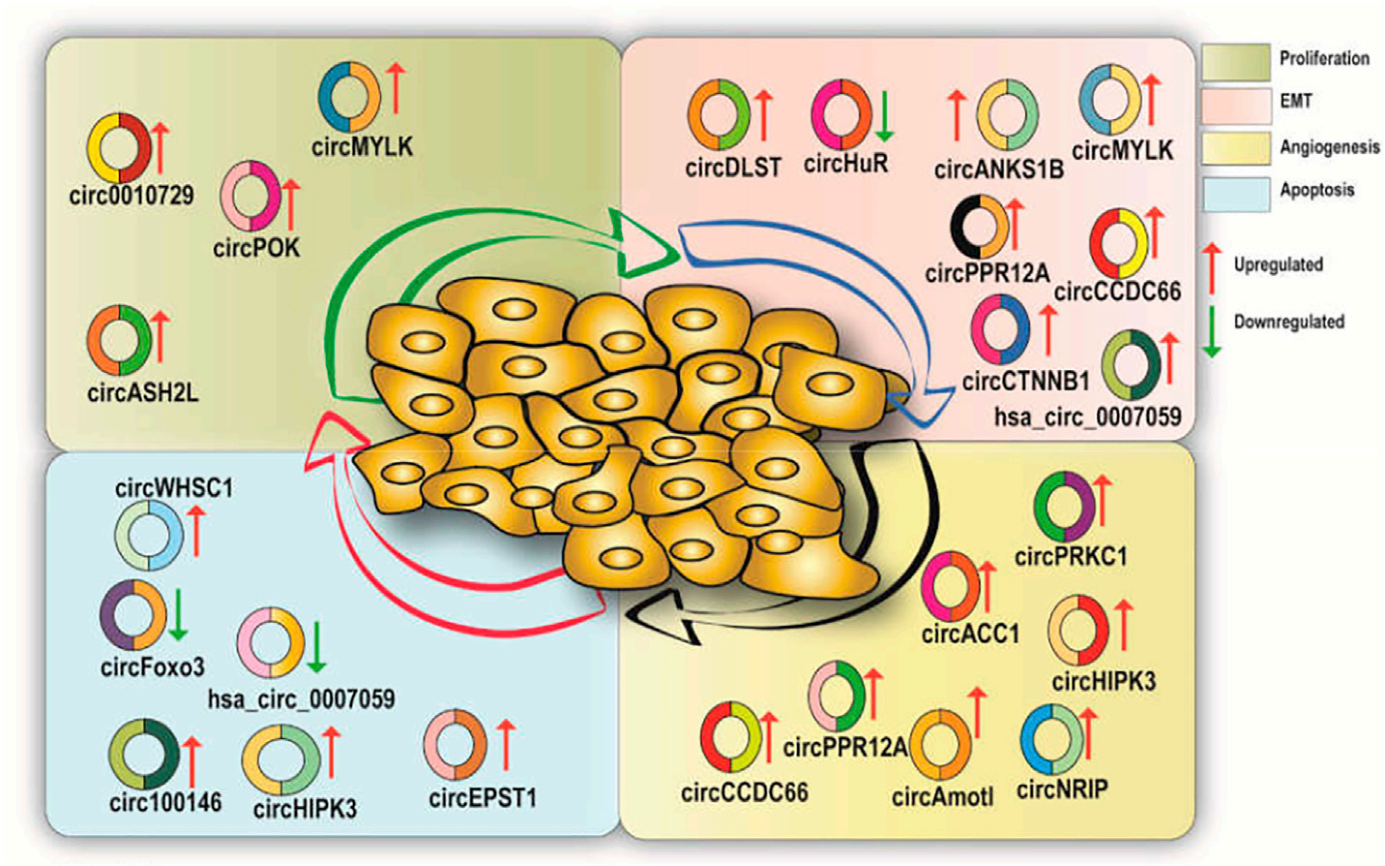
A diagrammatic illustration of the various circRNAs which are either upregulated or downregulated in various hallmarks of cancer.

CircRNAs show different expression patterns in various human cell types and play physiological roles such as normal cell propagation and hematopoiesis (Bachmayr-Heyda et al., 2015; Bonizzato et al., 2016). CircRNAs also play a role in cancer initiation, progression, and metastasis. Multiple studies have reported the role of circRNAs in numerous cancer types including gastric, colorectal, hepatic, lung, bladder, and prostate cancer (CaP) (Figure 2; Table 1) (Salzman et al., 2013; Bachmayr-Heyda et al., 2015; Bonizzato et al., 2016; Lü et al., 2017). Recently, a genome-wide circRNA microarray study has reported 1,155 aberrantly expressed circRNAs in breast cancer tissues where 715 circRNAs were found to be upregulated and 440 were found to be downregulated (Lü et al., 2017). Another circRNA microarray study in pancreatic ductal adenocarcinoma (PDAC) reported an upregulation of 209 circRNAs and downregulation of 142 circRNAs (Li et al., 2016).

**TABLE 1:** Differentially expressed circRNAs and their function vis-à-vis their effect on cancer progression.

S.No.	CircRNA	Functions	Cancer type	Expression	Effect on cancer	References
1	CircMTO1	Sponging miRNA	Hepatocellular carcinoma	Downregulated	Suppression	Han et al. (2017)
2	CircRNA-100338			Upregulated	Progression	Huang et al. (2017)
3	CircHIPK3			Upregulated	Progression	Chen et al. (2018a)
4	Circ-CDYL			Upregulated	Progression	Wei et al. (2019)
5	Circß-catenin	Act as a translational template		Upregulated	Progression	Liang et al. (2019)
6	CircNRIP1	Sponging miRNA	Gastric carcinoma	Upregulated	Progression	Zhang and Zhang et al. (2019)
7	CircPVT1			Upregulated	Progression	Chen (2016)
8	CircPTK2	Protein scaffolding	Lung carcinoma	Downregulated	Suppression	Wang et al. (2018a)
9	Circ-TCF25	Sponging miRNA	Bladder carcinoma	Upregulated	Progression	Zhong et al. (2016)
10	CircCCDC66	Sponging miRNA	Colorectal carcinoma	Upregulated	Progression	Hsiao et al. (2017)
11	CircNSUN2	Protein scaffolding		Upregulated	Progression	Chen et al. (2019)

In breast cancer tissues, circ-MYO9B has been reported to be overexpressed. Further, specific silencing of circMYO9B resulted in a suppression of cancer cell proliferation, invasion, and metastasis in MCF7 and MDA-231 breast cancer cells. Mechanistically, it has been reported that circMYO9B sponges miR-4316 and enhances FOXP4 levels thereby promoting breast cancer progression and highlighting the key role of circMYO9B/miR-4316/FOXP4 axis in breast cancer (Wang et al., 2018a; Zhang and Zhang et al., 2019). Califf, (2018) reported that downregulation of circITGA7 is associated with the progression of colorectal cancer (CRC). Further, the overexpression of circITGA7 resulted in the inhibition of CRC cell proliferation and metastasis both *in vitro* and *in vivo*. Kong et al. (2017) reported the circ-SMARCA5 to function as an oncogenic circRNA in CaP *via* promoting cell proliferation and inhibiting apoptotic cell death. Here, the silencing of circ-SMARCA5 in Du-145 CaP cells resulted in inhibition of cell proliferation and induction of apoptosis (Kong et al., 2017). All these shreds of evidence indicate the involvement of circRNAs in cancer progression and recommend that targeting of circRNAs for cancer therapeutics could be beneficial in combating this deadly disease.

## CircRNA-miRNA-TFs Axis in Cancer Progression and Suppression

**FIGURE 3:**
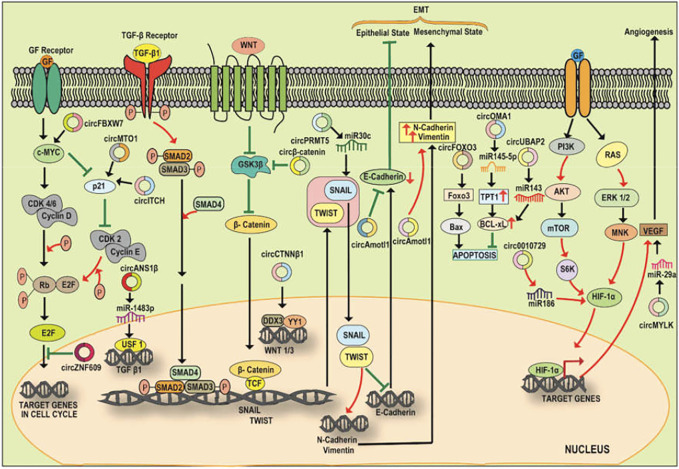
A diagrammatic illustration of the circRNA-miRNA-TF signaling axis in the progression or suppression of cancer. c-Myc, a cell cycle regulator is modulated by circMTO1 and circITCH *via* sponging miR-9, miR-17/miR-224 resulting in an enhanced p21 level and cell cycle progression. Increased expression of Circ-ZNF609 also promotes cell proliferation *via* c-Myc. The expression of pro-tumorigenic SNAIL and TWIST is also regulated by circRNA which interacts with the signaling pathways and promotes EMT. For instance, circPRMT5 *via* sponging miR-30c promotes the expression of SNAIL and TWIST which stimulate EMT *via* enhancing the expression of N-Cadherin/Vimentin and inhibiting E-CADHERIN. Circ-CTNNB1 binds to DDX3 facilitating the interaction of DDX3 with YY1 thereby transactivating YY1 and enhancing the expression of WNT1 and WNT3 which further bind to Frizzled receptor leading to amplification of Wnt/ß-Catenin pathway. Amplified Wnt/ß-Catenin signaling inhibits GSK3ß mediated destruction of ß-catenin, allowing translocation of cytoplasmic ß-Catenin to nucleus and initiation of transcription of the effectors involved in the process of EMT. circ ß-Catenin act as a decoy for GSK3ß and protects ß-Catenin from the GSK3ß mediated degradation resulting in the promotion of Wnt/ß-Catenin signaling and EMT in HCC. Circ-Foxo3 plays a pro-apoptotic role *via* sponging miR-22 and miR-138. Circ-UBAP2 and circOMA1 play anti-apoptotic roles by sponging miR-143 and decoying miR-145-5p respectively. The expression of HIF-1α is regulated by PI3K/AKT pathway. HIF-1α promotes the expression of VEGF thereby promoting angiogenesis. An upregulated circ_0010729 sponges miR-186 and inhibit the miR-186 mediated degradation of HIF-1α thus promoting angiogenesis. Circ-MYLK serves as a decoy for miR-29a and promotes the stability of VEGF resulting in angiogenesis.

Several studies have identified the sponging action of circular RNA on micro RNA (miRNA) as miRNA sponges as well as uncovered the circular RNA-miRNA-mRNA axis which activates pathways associated with various cancers (Sui et al., 2017). The network of circRNA, miRNA, and TFs play a role in the progression of cancer suggesting an undeviating link between the regulatory properties among the circRNA-miRNA-TF network (Sui et al., 2017). CircRNAs act as transcriptional regulators by sponging miRNAs and modulating the expression of the targets of miRNAs. For instance, cerebellar degeneration-related protein 1 antisense transcript (CDR1as) also termed as ciRS-7 (circular RNA sponge for miR-7) is the best-known circRNA which has been uncovered as a regulator of cellular processes and has over 70 binding sites for miR-7 (Hansen et al., 2013; Memczak et al., 2013). According to the competing endogenous RNA (ceRNA) hypothesis, CDR1as acts as a negative regulator of miR-7 (a widely studied tumor suppressor) and also influence several gene activities (Salmena et al., 2011; Hansen et al., 2013; Memczak et al., 2013; Tay et al., 2014) thereby making CDR1as a focus of cancer research (Kefas et al., 2008; Jung et al., 2012; Kong et al., 2012; Suto et al., 2015). For instance, CDR1as promotes colorectal cancer progression *via* sponging miR-7 and regulating EGFR-RAF1 activity (Weng et al., 2017). Further, it also acts as an inhibitor of miR-1299 and promotes breast cancer growth and metastasis (Sang et al., 2018). Another study by Zhong et al. (2016) reported that the interaction of circTCF25 with miR-103a-3p/miR-107 results in an upregulation of about 13 TFs playing role in cancer cell proliferation, migration, and invasion. Further, the overexpression of circTCF25 also leads to the downregulation of miR-103a-3p and miR-107 and subsequent upregulation of CDK6 resulting in cancer proliferation and migration. Thus, the above evidence suggests the modulatory role played by the circRNA-miRNA-TF axis in cancer progression (Figure 3; Table 2) and the prospective use of this axis as a biomarker and therapeutic target for cancer theranostics. Here, we summarize and discuss the role of some of the regulatory circRNA-miRNA-TFs axis in cancer progression, invasion, and migration.

**TABLE 2:** Regulatory role of the circRNA-miRNA-TFs axis in various aspects of carcinogenesis.

S.No.	CircRNA	Cancer type	miRNA	Target mRNA	Outcome	References
1	CircPTK2	NSCLC	miR-429/miR-200b-3p	TIF1 γ TGF-ß/SMAD	Inhibits cancer proliferation and EMT	Wang et al. (2018b)
2	CircHIPK3	Colorectal	miR-7	YY1	Promotes cancer progression	Zeng et al. (2018)
				EGFR	Inhibits cancer cell apoptosis	
				FAK		
3	CircANKS1B	Breast	miR-148a-3p/miR-152-3p	USF1	Promotes invasion, metastasis, and EMT	Zeng et al. (2018)
				TGF-ß		
				SMAD		
4	CircFBXW7	Glioblastoma	FBXW7-185aa	c-MYC	Inhibits proliferation and invasion	Yang et al. (2018a)
5	Circ-CDYL	HCC	miR-892a/328-3p	HDGF	Promotes cancer cell growth and propagation	Wei et al. (2020)
				HIF1AN		
				PI3K/AKT		
6	CircMTO1	HCC	miR-9	p21	Inhibits cancer progression	Han et al. (2017)
7	Circ-TCF25	Bladder	miR-103-3p/miR-107	CDK6	Promotes cancer progression	Zhong et al. (2016)
8	Crc-ITCH	Bladder	miR-17/miR-224	P21	Inhibits cancer progression	Yang et al. (2018b)
9	CircAmotl1	Prostate	miR-193a-5p	pcdha	Inhibition of EMT	Yang et al. (2019a)
10	Circ-Foxo3	Breast	miR-138	P53	Promotes apoptosis of cancer cells	Du et al. (2017)
11	Circ-HIAT1	Renal	miR-29c-3p/miR-195-5p/miR-29a-3p	CDC42	Promotes cancer invasion and metastasis	Wang et al. (2019)
12	CircNRIP1	Gastric	miR-149-5p	AKT/mTOR	Promotes cancer proliferation and invasion	Zhang and Zhang et al. (2019)
13	CircCCDC66	Colorectal	miR-33b/93	MYC	Promotes cancer growth, invasion, and metastasis	Hsiao et al. (2017)
14	CircPRMT5	Bladder	miR-30c	SNAIL	Promotes EMT and metastasis	Chen et al. (2018b)
15	CircUBAP2	Osteosarcoma	miR-143	Bcl-2	Promotes cancer proliferation	Zhang et al. (2017)
16	CircOMA1	Pituitary adenoma	miR-145-5p	TPT1	Inhibition of apoptosis and promotion of cancer progression	Du et al. (2019)
				Mcl-1		
				Bcl-xL		
17	Circ_001079	HUVEC	miR-186	HIF-1 α	Promotes cancer proliferation and angiogenesis	Dang et al. (2017)

### CircRNA-miRNA-C-MYC

The oncogenic MYC family has been implicated to be upregulated or aberrantly expressed in more than half of human cancers thereby playing an important role in cancer initiation and maintenance (Fatma and Siddique, 2020). c-MYC is one of the key TFs and accelerators of the cell cycle which plays a role in cell proliferation and is upregulated in various cancer types. Similarly, circRNAs have been reported to interfere with the regulatory cell cycle network leading to escape from cell cycle inhibitors and the generation of sustained proliferative signaling in several cancer types. Yang et al., 2017a reported that upregulated expression of Circ-Amotl1 promotes the growth and proliferation of breast cancer. Further, Circ-Amotl1 interacts with c-MYC and enables the retention and stability of c-MYC inside the nucleus and the resultant upregulation of c-MYC targets (Yang et al., 2017b). Another circRNA, circCCDC66 has been depicted to be aberrantly expressed in CRC. Here, an upregulation of circCCDC66 prevents the degradation of c-MYC mRNA *via* sponging miR-33b/miR-93 and resulting in the elevation of c-MYC levels and promotion of tumor cell growth and proliferation (Hsiao et al., 2017). Recently, Zhao et al. (2020) also reported an ectopic overexpression of circUHRF1 in oral squamous cell carcinoma. CircUHRF1 shows a sponging action towards miR-526b-5p which targets c-MYC expression. Thus, circUHRF1 regulates EMT progression of OSCC *via* the circUHRF1/miR-526b-5p/c-MYC axis. In another study on triple-negative breast cancer (TNBC), the upregulation of circ-PGAP3 was found to negatively correlate with miR-330-3p expression and positively correlate with the protein level of MYC. This study reported that circ-PGAP3 promotes TNBC growth and metastasis *via* sponging and inhibiting miR-330-3p and concomitant upregulation of c-MYC (He et al., 2020).

Circ-FBXW7 has also been reported to be involved in the reduction of c-MYC half-life *via* encoding a functional protein namely FBXW7-185aa. Mechanistically, the c-MYC protein level is maintained *via* the ubiquitin-proteasome pathway involving an E3 ligase FBXW7α and a ubiquitin-specific protease USP28. USP28 interact with the N-terminal domain of FBXW7α and results in antagonizing the c-MYC ubiquitylation and degradation. Interestingly, circ-FBXW7 encoded functional protein (FBXW7-185aa) shares the same 167 amino acid sequence with the N-terminal sequence of FBXW7α and therefore competes with FBXW7α in binding with USP28. The interaction of FBXW7-185aa with USP28 frees FBXW7α and leads to FBXW7α mediated degradation of c-MYC. Thus, a reduction in the expression of circ-FBXW7α will lead to an increase in the expression of oncogenic c-MYC and ultimate proliferation and invasion of glioblastoma (Yang et al., 2018a). circFBXW7 has also been reported to directly sponge miR-197-3p, mitigate the silencing of FBXW7 and inhibit the TNBC progression by increasing the abundance of FBXW7 and promoting the degradation of c-MYC (Ye et al., 2019). In HCC and bladder carcinoma, circRNA circMTO1 and circITCH have been reported to stabilize the levels of p21 mRNA (a downstream target of c-MYC) *via* sponging of miR-9/miR-17/miR-224, resulting in an increase in the level of p21. Thus, any downregulation in the levels of these circRNAs will result in the progression of HCC or bladder cancer (Han et al., 2017, Yang et al., 2018b).

### CircRNA-miRNA-TGF-ß/SMAD

TGF-ß signaling is actively involved in tumor metastasis and transition of Epithelial state to mesenchymal state (EMT) in various human cancers (Kaminska and Cyranowski, 2020). EMT is under the regulatory role of upstream signaling pathway viz. TGF-ß/SMAD**.** CircRNAs have been reported to interfere with EMT and tumor metastasis *via* participating in the TGF-ß signaling. For instance, circANKS1B has been reported to be upregulated in TNBC where it sponges miR-148a-3p/miR-152-3p to enhance the expression levels of a TF, USF1 which further results in upregulating TGF-ß expression and then TGF-ß/SMAD regulated EMT (Zeng et al., 2018). On the contrary, circPTK2 has also been reported to sequester miR-429/miR-200b-3p and enhance the expression of TIF1γ resulting in inhibiting the TGF-ß/SMAD pathway and EMT process. Here, a decrease in the expression of circPTK2 promotes invasion and metastasis in non-small cell lung cancer (NSCLC) (Wang et al., 2018b). In a study, Zhang et al. (2018a) investigated the relationship between circSMAD2 and HCC cells/tissues. The group observed that circSMAD2 was downregulated in HCC tissues and this downregulation correlates with the level of tumor differentiation where lower levels of circSMAD2 were found in the poorly differentiated HCC tissues. SMAD2, the gene from which circSMAD2 is generated plays a key role in EMT and HCC progression (Heldin and Moustakas, 2012). Further, the upregulation of circSMAD2 is also observed during the TGF-β induced EMT (Conn et al., 2015). In contrast to this, Zhang et al. (2018b) observed that upregulated circSMAD2 in HCC cells inhibited migration, invasion, and EMT. Here, circSMAD2 sponges miR-629, a miRNA involved in EMT *via* downregulating the tripartite motif-containing 33 (TRIM33) expression and promoting the TGF-β/SMAD2 signalling. Thus, circSMAD2 sponges miR-629 and inhibit the invasion, migration, and EMT in HCC cancer cells *via* the TGF-β/SMAD2 signalling (Zhang et al., 2018d). However, the study was *in vitro* and requires further *in vivo* validation.

Huang et al. (2020a) introduced the involvement of circRNA, cESRP1 in small cell lung cancer (SCLC). They reported for the first time that cESRP1 plays a regulatory role in chemosensitivity of SCLC *via* sponging miR-93-5p-SMAD7/p21 axis and inhibiting TGF-ß mediated EMT in SCLC. Another circRNA viz circ-OXCT1 has been reported to be downregulated in GC cell lines and tissues and this downregulation is associated with lymph node metastasis and survival rate (Liu et al., 2020). Further, the data suggest that overexpression of circ-OXCT1 in gastric cancer suppresses EMT and metastasis *via* the TGF-ß/SMAD signaling pathway. Here, knockdown of circ-OXCT1 downregulated the expression of SMAD4 *via* sponging miR-136 and consequently regulated the levels of E-Cadherin, N-Cadherin, and Vimentin *via* the TGF-ß/SMAD signaling pathway resulting in promoting EMT, and cancer (Liu et al., 2020). Thus, the clinicopathological studies and data suggest the circ-RNA-TGF-ß axis as a potential target for treating advanced-stage carcinoma. Further, Shang et al. (2020) reported the circPACRGL mediated progression of CRC cells through the miR-142-3p/miR-506-3p/TGF-ß axis. The group reported that cancer-derived exosomal circPACRGL acts as a miRNA sponge for miR-142-3p/miR-506-3p and stimulate the expression of TGF-ß1 in CRC cell proliferation, invasion, and migration. Xu et al. (2020) reported another circRNA, CCDC66 which is upregulated in gastric cancer tissues and this upregulation correlates with the concomitant triggering of the TGF- ß pathway and tumor progression. Further, the knockdown of circCCDC66 resulted in inducing programmed cell death and suppressing gastric cancer growth.

### CircRNA-miRNA-SNAIL/TWIST

SNAIL and TWIST are the principal transcription factors in the TGF-ß/SMAD and Wnt/ß-Catenin signalling pathways. SNAIL and TWIST are critical EMT-inducing TFs that increase the expression levels of Vimentin (Mahmood et al., 2017). Recently, circRNAs have been reported to crosstalk with these TFs involved in the EMT process. For instance, in bladder urothelial carcinoma, circPRMT5 is reported to be involved in the promotion of EMT and cancer cell metastasis *via* sponging and elevation of miR-30c and SNAIL, respectively (Shibue and Weinberg, 2017; Chen et al., 2018b). circMET is another circRNA that is upregulated in HCC tumors and the aberrant expression of circMET correlates with the tumor recurrence in HCC patients. Mechanistically, circMET overexpression promotes EMT and result in lowering the immune response by inducing an immunosuppressive microenvironment in HCC patients through the miR-30-5p/Snail/(DPP4)/CXCL10 axis (Huang et al., 2020b). Mechanistically, DPP4, a downstream target of SNAIL is involved in immune suppression *via* negative regulation of the lymphocyte trafficking. DDP4 cleaves CXCL10 chemokine which is important in determining the trafficking of effector T-cells to the tumor microenvironment. The inhibition of DPP4 preserves the CXCL10 chemokine and enhances the tumor immunity and the migration of T-cells to the tumor microenvironment. Thus, the study revealed that circMET acts as an onco-circRNA *via* the miR-30-5p/SNAIL/DPP4/CXCL10 axis. (Hollande et al., 2019). Guo et al. (2019) reported an upregulated circ-ZNF652 acting as a prognostic marker for HCC patients. Circ-ZNF652 increases the expression of EMT trigger SNAIL *via* the sponging of miR-203/miR-502-5p thereby promoting metastasis in HCC. Further, the knockdown of circ-ZNF652 led to a reduction in the metastatic capability of HCC cells both *in vitro* and *in vivo* conditions*.* Mechanistically, the circuitry involved here includes SNAIL which binds directly to the E-box motif (CAGGTG) present on the promoter of circ-ZNF652 thereby enhancing circ-ZNF652 levels (Guo et al., 2019). Thus, the circ-ZNF652/miR-203/miR-502-5p/SNAIL axis would be a potential target for HCC management and treatment. circFNDC3B-218aa has also been depicted to play an inhibitory role in the progression of CRC. Here, circFNDC3B-218aa inhibits the expression of SNAIL and subsequently upregulates FBP1 expression thereby inhibiting EMT in CRC and acting as a therapeutic target for CRC (Pan et al., 2020). CircRNA_0084043 is another circRNA that is significantly upregulated in melanoma tissue and promotes the cancer progression and metastasis *via* directly binding and sponging miR-153-3p and leading to the upregulation of SNAIL thereby functioning as an oncogene in melanoma (Luan et al., 2018).

TWIST has also been reported to regulate the expression of circRNA in the induction of EMT. TWIST binds and activates the transcription of the Cul2 promoter leading to the expression of Cul2 circular RNA (circ-10720) and the resultant sponging of miR-490-5p, a miRNA involved in targeting and suppressing Vimentin (an EMT marker). Thus, TWIST indirectly leads to the induction of EMT *via* the cul2/miR-490-5p axis (Meng et al., 2018). Similarly, circAMOTL1L has been reported to upregulate E-cadherin and downregulate Vimentin resulting in the inhibition of EMT and CaP progression (Yang et al., 2019a).

### CircRNA-miRNA-FOXO3

Forkhead box 3 (FOXO3) protein belongs to the FOXO subclass of TF. FOXO3 mediates several biological processes including cell propagation, DNA damage, apoptosis, and tumorigenesis (Liu et al., 2018a). FOXO3 also leads to the induction of apoptosis *via* upregulation of PUMA (a pro-apoptotic protein). It has been reported that circRNA i.e. circ-FOXO3 performs a proapoptotic role *via* promoting FOXO3 expression (Du et al., 2017). Mechanistically, circ-FOXO3 forms a complex with p53 (tumor suppressor gene) and MDM2 resulting in the aggravation of MDM2-induced degradation of p53 and protecting FOXO3 from MDM2 dependent degradation. Later, FOXO3 leads to the upregulation of its downstream effector proteins including pro-apoptotic proteins, such as PUMA and BAX leading to the apoptosis of tumor cells (Du et al., 2017). Circ-FOXO3 also performs this proapoptotic role *via* absorbing and sponging miR-22 and miR-138. These miRNAs are involved in targeting the FOXO3 mRNA (Yang et al., 2016). In contrast, circUBAP2 and circOMA1 also direct the upregulation of anti-apoptotic proteins or BCL-2 family proteins and result in inhibiting programmed cell death (Zhang et al., 2017; Du et al., 2019). Mechanistically, circUBAP2 sponges miR-143 and enhance the expression of BCL-2 in the case of osteosarcoma (Zhang et al., 2017). Similarly, circOMA1 decoys miR-145-5p and inhibit the suppression of TPT1 (a regulator of cancer stem cell components) by miR-145-5p. The upregulated TPT1 then leads to the upregulation of downstream effector proteins, MCL-1 and BCL-xL in pituitary adenomas (Du et al., 2019). In NSCLC, circ-FOXO3 plays an anti-oncogenic role *via* sponging miR-155 and elevating the FOXO3 levels. Further, downregulation of circ-FOXO3 was found to correlate with several aspects of carcinogenesis such as cell proliferation, invasion, and migration of NSCLC cells (Zhang et al., 2018c).

In HCC, circFBXO11 has been insinuated in the sustained cellular proliferation and progression *via* the circFBXO11/miR-605/FOXO3 axis. The cirFBXO11/miR-605/FOXO3 axis has ABCB1, a multidrug resistance protein, MDR1 as its downstream target. Thus, this axis regulates tumorigenesis and chemoresistance in HCC (Li et al., 2020). Similarly, a high-throughput microarray assay with the samples of gastric cancer patients has reported the aberrant expression of circRNA hsa_circ_0001368 in gastric cancer both *in vitro* and in *an* animal model. This study suggested that hsa_circ_0001368 has the potential of sponging miR-6506-5p and this sponging leads to elevating the expression of the tumor-suppressive gene, FOXO3 (associated with apoptosis of tumor cells). Further, a knockdown study on hsa_circ_0001368 revealed that knockdown of this circRNA derives cancer progression in gastric cancer cells both *in vitro* and *in vivo* (Lu et al., 2019b).

### CircRNA-miRNA-ß-Catenin

ß-Catenin is a multifunctional TF that is associated with the canonical Wnt signaling pathway. Wnt/ß-Catenin is a major pathway involved in the progression of multiple cancers. Recently, the interaction of circRNAs with Wnt/ß-Catenin pathways has been reported where circRNAs regulate this signaling pathway and modulate the progression of cancer. For instance, circ-ITCH *via* sponging oncogenic miRNAs suppress the WNT/ß-Catenin signaling pathway (Wan et al., 2016). Further, in esophageal squamous cell carcinoma (ESCC), circ-ITCH perform a sponging action against miR-7, miR-17, and miR-214. The upregulation of circ-ITCH results in ubiquitination and degradation of phosphorylated DVL-2 (disheveled homolog DVL-2) thereby inhibiting the Wnt/ß-Catenin pathway and suggesting the inhibitory effect of circ-ITCH on ESCC (Li et al., 2015b). Circ-CTNNB1 is another aberrantly expressed circRNA in several cancer types such as gastric and colon cancer (Yang et al., 2019b). Circ-CTNNB1 interacts with Dead-box polypeptide 3 (DDX3) facilitating the interaction of DDX3 with Yin Yang 1 (YY1) TF bringing about the transactivation of YY1 and increasing the expression of downstream genes such as WNT1 and WNT3. The overexpressed WNT1 and WNT3 further interact with the Frizzled receptor resulting in the amplification of the Wnt/ß-Catenin signaling pathway. The amplified Wnt/ß-Catenin signaling results in the inhibition of GSK3ß mediated destruction of ß-catenin, allowing translocation of the ß-Catenin from the cytoplasm to the nucleus, and commencing the transcription of effectors playing part in the process of EMT (Yang et al., 2019a).

Circß-Catenin, a cytoplasmically localized circRNA is derived from the ß-Catenin gene locus and is highly expressed in the liver cancer tissues. It has been reported to act as a decoy for GSK3ß and protects ß-Catenin from the GSK3ß mediated degradation resulting in the promotion of Wnt/ß-Catenin signaling and EMT in HCC (Liang et al., 2019). Further, circ_0067934 has been reported to support cancer progression and migration in Hep3B and Huh7 HCC cells *via* inhibiting miR-1324 and activating FZD5 mRNA and Wnt/ß-Catenin signaling pathway. Thus, circ_0067934/miR-1324/FZD5/ß-Catenin axis could be used as a therapeutic target for HCC management (Zhu et al., 2018). Similarly, circ-CBFB acts as a miR-607 sponge which is an inhibitor of FZD3. Thus, circ-CZFB sponges miR-607 and promotes FZD3 expression resulting in activating the Wnt/ß-Catenin pathway and the consequent progression of chronic lymphocytic leukemia (Xia et al., 2018). Additionally, some other circRNAs have been disclosed to modulate the Wnt/ß-Catenin pathways. These include circRNA_102171, circ_0067934, and circRNA_100290 which activate the Wnt signaling pathway by sponging CTNNBIP1, miR-1324, and miR-516 and thereby promoting the progression, invasion, and metastasis of thyroid, liver, and CRC, respectively (Bi et al., 2018; Fang et al., 2018; Zhu et al., 2018). In contrast, circ_0006427 inactivates the Wnt signaling pathway through sponging miR-6783-3p and consequently suppressing the progression of lung adenocarcinoma (Yao et al., 2019).

### CircRNA-miRNA-HIF-1α

Hypoxia-inducible factor-1α (HIF-1α) is another important transcription factor that functions in response to hypoxic conditions and promotes the expression of genes related to angiogenesis such as vascular endothelial growth factor (VEGF), a predominant growth factor involved in promoting the endothelial cell proliferation to form blood vessels (Apte et al., 2019). HIF-1α has been recently reported to be regulated by circRNAs. Dang et al. (2017) reported that circ_0010729 is upregulated under hypoxic conditions and sponges miR-186 to inhibit the miR-186 regulated degradation of HIF-1α thereby allowing the HIF-1α expression and facilitating angiogenesis. The circ-HIPK3 is another circRNA that acts as a competing endogenous RNA and functions in cancer progression and metastasis *via* sponging miR-338-3p and enhancing the HIF-1α levels resulting in HIF-1α mediated EMT of cervical cancer (Qian et al., 2020). Another study has reported that circRNA_100859 is significantly overexpressed in colon cancer and functions as an oncogene in the proliferation of colon cancer. The use of the circNET database in this study revealed that miR-217 is a target of this circRNA. Circ_100859 sponges miR-217 which further directly targets the HIF-1α gene and promotes colon cancer progression *via* this circ_100859/miR-217/HIF-1α axis (Zhou et al., 2020). In HCC, hsa_circ_0046600 is highly upregulated and this upregulation is found to correlate with the tumor size and vascular invasion in HCC patients. This has been reported to occur *via* the circRNA/miRNA/TF axis. Here, hsa_circ_0046600 sponges and inhibits miR-640 thereby promoting the expression of HIF-1α and progression of HCC (Zhai et al., 2019). The above evidence suggests the emerging role of circRNA as cancer biomarkers and the potential of the circRNA-miRNA-TFs axis as a therapeutic target for cancer management.

## CircRNA-miRNA-TF Axis Based Targeted Therapeutic Strategies for Cancer Treatment

The circRNA-miRNA-TFs axis has a crucial role in the progression or suppression of cancer. As circRNA has several miRNAs binding sites, targeting and inhibiting circRNAs may serve as a potential and more beneficial therapeutic approach than targeting or inhibiting a single miRNA/gene. The most common approach for determining the functions of circRNA is RNA interference (RNAi). RNAi follows a loss-of-function approach to determine the functions of circRNA. For this purpose, the viral vector packaged circRNA transcripts could be delivered to the target cells to explore their therapeutic effects (Huang et al., 2019). The inhibition of the specific circRNA will help in enhancing the protective action of the miRNA in the inhibition of oncogenes such as β-catenin (Xia et al., 2018; Shang et al., 2019; Chen et al., 2020a; Chen et al., 2020b). Further, the CRISPR/Cas9 mediated technology of genetic engineering also serves as a novel tool in the investigation of circRNAs. The CRISPR/Cas-mediated homologous recombination may lead to the replacement of a circRNA gene with a marker gene thereby resulting in the consumption of circRNA without affecting the existing gene (Ho et al., 2015). For this purpose, the investigation and screening of circRNA with libraries equipped with distinct guide RNA (gRNA) will help in boosting targeted therapies.

The current targeted strategy for circRNA could involve the synthesis of a circRNA sponge using the approach of simple enzymatic ligation. The artificially synthesized circRNA molecule could be exogenously applied as a miRNA inhibitor to facilitate the binding as well as blocking of miRNA maturation thereby serving as a propitious cancer management approach (Liu et al., 2018b). In a study, Jost et al. (2018) artificially engineered the miR-122 sequestering circRNA sponges *in vitro* and isolated miR-122 from the Hepatitis C virus (HCV). Further, circRNAs can also be exploited in sponging proteins. The bindings sites procured from CLIP and SELEX data can be utilized for many RBPs (Jost et al., 2018). The circular miR-122 RNA sponge against HCV could be employed in combination with the sequences that sequester the host factors (hnRNP L and HuR) which are required for the propagation of HCV. Thus, the artificially engineered circRNA sponge may function as a promising tool in circRNA-based targeted anti-tumor therapy and has a potential application in molecular medicine and therapeutics.

Exosomes are also a burning topic in the field of cancer-based research and therapy, which serves as a delivery vehicle for circRNA, lncRNA, mRNA, miRNA, and other proteins and lipids (Shi et al., 2020). Furthermore, the roles of exosome-derived circRNAs have also been reported to be used as biomarkers in clinical applications (Wang et al., 2020a). They usually range from 30 to 100 nm in diameter. Importantly, neoplastic cells secrete a great number of exosomes (10 times) than other cells of the biological system (Xiong et al., 2019). In a study by Wang and their team, exosomes derived from CRC cells upregulated PKM2 *via* microsponging of miR-122 by ciRS-122 circRNA. The up-regulation of PKM2 releases more energy (due to liberation of ATP and glycolysis) for transporters which eject the drug out of the cell. It was also found that ciRS-122 delivered *via* exosomes to non-chemo resistant cells enhances the drug resistance. Taking advantage of this study, exosomes based delivery of siRNA to ciRS-122 circRNA was designed that elevate the level of miR-122 and decrease the level of PKM2 influencing the sensitivity of oxaliplatin (a platinum-based chemotherapeutic agent) in mice (Wang et al., 2020b). Exosomes could also transmit circ_0051443 from normal to HCC cells resulting in inhibiting the malignant nature of HCC *via* the induction of programmed cell death and cell cycle arrest (Chen et al., 2020). Here, circ-0051443 reduced the tumor size and volume *via* the upregulation of BAK1.

Interestingly, emerging shreds of evidence have portrayed the therapeutic function of circRNA encoded tumor-related functional peptides. These functional peptides include cancer-inhibiting proteins or peptides such as circ β-Catenin encoded β-Catenin-370aa; circ-AKT3 encoded AKT3-174aa; circPPP1R12A encoded circPPP1R12A-73aa (Wu et al., 2020). These functional peptides encoded by circRNAs play predominant roles in carcinogenesis making them novel targets for cancer treatment and drug development (Wu et al., 2020). The clinical significance of these circRNA encoded functional peptides enable the use of these peptides in the treatment of cancer shortly. For this purpose, an appropriate delivery system is required for the transportation of both artificially engineered circRNA and the functional peptides to the target cell. The emerging field of nanomedicine and the development of nanoparticles-based preparations could improve the intracellular entry of therapeutic cargos (Khan et al., 2021). Nanoparticles could be used as an efficient drug delivery molecule for the treatment and management of cancer in many ways such as through intravenous and tail vein injection thereby serving as a promising tool for therapeutic purposes. A recent plasmid-based delivery system has been established by Yi et al. (2020) viz. the Micropoly-transfecter which is capable of delivering circ-1073 plasmid *via* intratumoral injection resulting in the inhibition of tumor development. Presently, the circRNA mediated targeted cancer therapy is still in its beginning stage, and therefore more extensive research needs to be done to unfold the use of the circRNA-miRNA-TF axis as a therapeutic target.

## Nanomedicine: A Hope to Target the CircRNA-miRNA-TF Axis

Targeted therapies have gained widespread attention in cancer management (Ke and Wu, 2016). However, the demand for safe and 100 percent effective treatment is still a lag. The development of nanoparticles-based drug delivery molecules could improve the delivery of circRNA based therapeutic cargos. Through *in vivo* studies, nanoparticle-based delivery systems have elevated the feasibility of circRNA. Recently, Du et al. (2018) designed a bifunctional therapeutic approach against breast cancer. Gold nanoparticles conjugated with siRNA targeting CircDnmt1 or anti-sense oligonucleotide (AON) targeting binding site over circDnmt1 meant for p53 and Auf1 was constructed, which resulted in the suppression of tumor cells and enhanced cellular autophagy and survival of mice (Du et al., 2018; Zhang et al., 2020). Furthermore, the deliverance of circFOXO3 plasmid through gold nanoparticles helped in the repression of tumor metastasis (Du et al., 2017). In another study, the tumor growth and mouse survival were increased after the treatment with targeted delivery of gold nanoparticles for AON blocking binding site on circCCNB1 for CDK1 and CCNB1 (Fang et al., 2019). Thus, from these studies, we speculate that nanoparticles-based delivery of circRNA-miRNA based therapy could provide a new way of treatment to chemoresistant and non-chemoresistant cancer. However, it further requires more extensive studies using various cancer animal models (Khan et al., 2019).

## Conclusion and Future Perspective

In the past few years, circRNAs have evolved as a new puzzle and a focus of research in cancer biology and therapy. Despite great progress in the characterization of circRNA, many factors still exist to be explored. Firstly, cancer-related available database RNA-seq data sets are put together applying a poly-A purification step to specifically enrich the mRNAs. This purification step reduces the possibility of identification of circRNAs that lack a poly-A tail (Kristensen et al., 2018). Next, circRNA discovery involves circRNA detection and quantification which might be affected by template switching artifacts (which mimics linear splicing and back splicing) and rolling circle amplification during RT-PCR (Szabo and Salzman, 2016). Some studies reported that circRNAs dysregulations in cancer are due to abnormal biogenesis. Studies need to be done to identify the detailed mechanism of production and turnover of circRNA which will contribute to understanding the circRNA dysregulation in cancer. Further, miRNA sponging has been considered a common mechanism of action of circRNA. For instance, CRD1as and circular Sry RNA function as efficient miRNA sponges in the regulation of gene expression. However, this sponging action faces challenges as few circRNAs harbor numerous miRNA binding sites for one miRNA such as in the case of CDR1as and circular Sry RNA (Guo., 2014). Further, the abundance of several circRNAs is lower than that of miRNAs making it difficult to achieve the miRNA sponging effect. Thus, the stoichiometric relevance between the miRNA-binding site of the sponge and the ultimate miRNA target site should be carefully taken into consideration. For the validation of the proposed sponging functioning, Ago-CLIP and luciferase reporter assays would be beneficial. Next, some studies conflict with the miRNA sponging action of circRNAs. For instance, Kristensen et al. (2020) reported that the putative oncogenic ciRS-7 that is abundant in tumors is not expressed in the colon tumor cells but the stromal cells within the tumor microenvironment relative to the uninvolved stromal cells. The high ciRS-7 expression in the tumor stromal cells is correlated with poor prognosis. Further, the correlation established between circRNA and mRNA expression which is generally interpreted as evidence of ceRNA function is also controversial. ciRS-7 correlates with the expression of miR-7 target genes depending on whether they are co-expressed or mutually exclusive in cancer or stromal cells (Kristensen et al., 2020). Therefore, extensive and more challenging studies on circRNAs need to be done to carefully establish the exact functioning and expression patterns of circRNAs in cancer progression.

Further, most of the circRNAs are lowly expressed and are unlikely to have functional relevance. A group of researchers has reported that there is no association of circRNAs with polysomes (Guo et al., 2014; You et al., 2015), arguing against the notion that circRNAs function through their protein products (Kristensen et al., 2019). Further, the translation of circRNA depends on IRES, but IRES is present only in <1.5% circRNAs (Fan et al., 2019). It is noteworthy that circRNA translation also does not confirm its advantage as translation could itself be an error due to fallacious initiation and the resultant protein product could be functionless or malignant. Therefore, extensive studies on establishing the functional relevance of circRNAs and their products need to be done. Moreover, most of the differentially expressed circRNAs in cancer may not necessarily be cancer drivers. For instance, the brain shows more back-spliced genes and has a higher back slicing rate than the heart, kidney, liver, or any other organ (Xu and Zhang., 2021). This observation however does not necessarily support the proposal that circRNAs play important roles in the brain (Rybak-Wolf et al., 2015). In the brain, the rate of back-splicing is still very low and the fraction of deleterious back-splicing is atleast 73.2% (Xu and Zhang., 2021). Further, brain cells have a longer life span than any other cells leading to the accumulation of circRNAs at a higher level than other tissues. Taken together, back-splicing events may be splicing errors. Therefore, circRNAs produced by back-splicing events may be functional or non-functional and the aberrant or high expressions of circRNAs do not necessarily indicate their relevance in cancer progression.

Next, if the balance between the circularisation, degradation, and localization of circRNA is not fine-tuned, the expression of circRNA will be disturbed leading to cancer progression. Ultimately, the main focus of research should be on how circRNAs could be utilized clinically in cancer treatment. circRNAs have shown varying expression patterns in cancer patients suggesting circRNAs as promising diagnostic and prognostic markers. However, the discovery of circRNAs is still in its juvenile phase and more elaborative studies should be done for the screening of circRNAs in cancer samples. The past decade has also observed the role of natural products and phytochemicals in the treatment of several diseases including cancer (Singh et al., 2019; Singh et al., 2021b). In addition, the role of phytochemicals in the modulation of ncRNAs involved in chronic diseases has also been reported (Zhang et al., 2021). Therefore, the development of phytochemical-based novel strategies to target circRNAs could be beneficial in managing this disease. Also, a detailed understanding of the mode of action of circRNAs and the circRNA mediated modulation of miRNA and other downstream targets need to be focused on. With more technological advancements and a precise understanding of the circRNA-miRNA-TF regulatory axis, the advancement of circRNA based cancer therapeutics will be boosted and this will help in solving the confusing carcinoma puzzle a little bit more. In this direction, nano-enabled strategies could play an important role. In conclusion, this comprehensive review will contribute to enhancing the present-day knowledge related to the circRNA-miRNA-TF axis in cancer pathogenesis and development.

## Abbreviations

ABCB1, ATP Binding Cassette Subfamily B Member 1; AMPK, AMP-Activated Protein Kinase; CDC42, Cell Division Cycle 42; CDK6, Cyclin-Dependent Kinase 6; CN-AML, Cytogenetically Normal Acute Myeloid Leukemia; CRISPR, Clustered Regularly Interspaced Short Palindromic Repeats; CTNNBIP1, Catenin Beta Interacting Protein 1; CXCL10, C-X-C Motif Chemokine Ligand 10; DPP4, Dipeptidyl-Peptidase 4; FBP1, Fructose-Bisphosphatase 1; Hel25E, Helicase at 25E; HIPK3, Homeodomain Interacting Protein Kinase 3; HUVEC, Human Umbilical Vein Endothelial Cells; PABPN1, Polyadenylate-Binding Protein, Nuclear 1; TPT1, Translationally-Controlled Tumor Protein 1; YTHDC1, YTH Domain-Containing protein 1; YTHDF3, YTH N6-Methyladenosine RNA Binding Protein 3.
